# Impacts of the Affordable Care Act on Receipt of Women’s Preventive Services in Community Health Centers in Medicaid Expansion and Nonexpansion States

**DOI:** 10.1016/j.whi.2020.08.011

**Published:** 2020-10-03

**Authors:** Brigit Hatch, Megan Hoopes, Blair G. Darney, Miguel Marino, Anna Rose Templeton, Teresa Schmidt, Erika Cottrell

**Affiliations:** a School of Medicine, Family Medicine, Oregon Health & Science University, Portland, Oregon; b OCHIN, Inc, Portland, Oregon; c School of Medicine, Obstetrics and Gynecology, Oregon Health & Science University, Portland, Oregon

## Abstract

**Background:**

The Affordable Care Act (ACA) increased health insurance coverage throughout the United States and improved care delivery for some services. We assess whether ACA implementation and Medicaid expansion were followed by greater receipt of recommended preventive services among women and girls in a large network of community health centers.

**Methods:**

Using electronic health record data from 354 community health centers in 14 states (10 expansion, 4 non-expansion), we used generalized estimating equations and difference-in-difference methods to compare receipt of six recommended preventive services (cervical cancer screening, human papilloma virus vaccination, chlamydia screening, influenza vaccination, human immunodeficiency virus screening, and blood pressure screening) among active female patients ages 11 to 65 (*N* = 711,121) before and after ACA implementation and between states that expanded versus did not expand Medicaid.

**Results:**

Except for blood pressure screening, receipt of all examined preventive services increased after ACA implementation in both Medicaid expansion and nonexpansion states. Influenza vaccination and blood pressure screening increased more in expansion states (adjusted absolute prevalence difference-in-difference, 1.55; 95% confidence interval, 0.51–2.60; and 1.98; 95% confidence interval, 0.91–3.05, respectively). Chlamydia screening increased more in nonexpansion states (adjusted absolute prevalence difference-in-difference: −4.21; 95% confidence interval, −6.98 to −1.45). Increases in cervical cancer screening, human immunodeficiency virus screening, and human papilloma virus vaccination did not differ significantly between expansion and nonexpansion states.

**Conclusions:**

Among female patients at community health centers, receipt of recommended preventive care improved after ACA implementation in both Medicaid expansion and nonexpansion states, although the overall rates remained low. Continued support is needed to overcome barriers to preventive care in this population.

Rates of health insurance coverage increased dramatically in 2014 following implementation of insurance expansion provisions under the Affordable Care Act (ACA) (Kominski, Nonzee, & Sorensen, 2017). A new public mandate required enrollment in health insurance, and individuals could enroll in health insurance through the new marketplace of private health insurers or through state Medicaid programs for which the federal government funded expansion to 138% of the federal poverty level (FPL). Although federally funded Medicaid expansion was available to all states, many opted against ACA-sponsored expansion and chose to maintain current eligibility criteria in their states (Kaiser Family Foundation, 2020). Among women, the rate of uninsurance decreased from 19% in 2013 to 11% in 2017 (Kaiser Family Foundation, 2018). This trend was consistent in states that expanded Medicaid and those that did not, although in Medicaid expansion states more patients gained Medicaid insurance specifically (Angier et al., 2017; Courtemanche, Marton, Ukert, Yelowitz, & Zapata, 2018; Huguet et al., 2017).

The ACA also increased financial support to federally qualified health centers and other organizations using the community health center (CHC) model to provide care to individuals with no health insurance; these centers serve the majority of patients with Medicaid insurance in the United States (Department of Health and Human Services, 2017; Tilhou, Huguet, DeVoe, & Angier, 2020). Historically, CHCs have served a large proportion of women, with some estimates suggesting that CHCs serve one in five low-income women of reproductive age in the United States (Wood et al., 2013). After ACA implementation, CHCs saw an influx of new patients and also continued serving a large population of patients with no health insurance (Angier et al., 2015; Antonisse, Garfield, Rudowitz, & Guth, 2019; Han, Luo, & Ku, 2017; Hoopes et al., 2016). New patients to CHCs tended to be younger and suffer from more mental and physical comorbidity compared with previously established patients (Hatch et al., 2018)―shifting demographics that can impact the care of all patients.

In addition to expanding insurance and financial support for CHCs, the ACA specified coverage without cost-sharing for preventive services (U.S. Government, 2015), including women’s preventive health services (Agency for Healthcare Research and Quality, 2015). Despite evidence that preventive care is linked to improved health, decreased morbidity and mortality, and high cost effectiveness (Maciosek, Coffield, Flottemesch, Edwards, & Solberg, 2010), many women of reproductive age do not receive recommended preventive care (Pazol et al., 2017; Stolp & Fox, 2015). Previous studies have demonstrated the association between health insurance and increased receipt of recommended health care (Bailey et al., 2015) and preventive services (Cowburn, Carlson, Lapidus, & DeVoe, 2013; DeVoe, Fryer, Phillips, & Green, 2003; Marino et al., 2016). Since the implementation of the ACA, early studies suggest an increase in receipt of preventive services and other recommended health care (Huguet et al., 2018; Sabik & Adunlin, 2017; Sun, Cole, Lipsitz, & Trinh, 2018). A few studies compare these trends in Medicaid expansion states to those in nonexpansion states and find few differences in receipt of care (Alharbi, Khan, Horner, Brandt, & Chapman, 2019; Huguet et al., 2019). However, less is known about how ACA implementation has impacted women’s preventive care, and one study showed young women had limited understanding of these new benefits, particularly if they had less education, had lower income, and were uninsured (Hall, Fendrick, Zochowski, & Dalton, 2014). It is critical to assess the impact of ACA implementation on receipt of preventive care among reproductive-aged women, particularly those from low-income and uninsured populations served by CHCs.

The purpose of this study, which used electronic health record (EHR) data from a multistate network of CHCs, was to 1) assess the change in receipt of preventive care among women and girls 2 years before versus 2 years after ACA implementation and 2) compare pre–post changes in the receipt of care between Medicaid expansion versus nonexpansion states. We hypothesized that in this CHC clinic population serving largely low-income patients, preventive care would increase after ACA implementation, with greater gains among states that expanded Medicaid relative to those that did not.

## Methods

To test our hypotheses, we conducted a patient-level cross-sectional analysis comparing CHC patients during two time periods: the 2 years before ACA insurance expansion (2012–2013) and the 2 years afterward (2014–2015).

### Data Source

We used EHR data from the Accelerating Data Value Across a National Community Health Center Network (ADVANCE) clinical research network, a member of PCORnet (Corley, Feigelson, Lieu, & McGlynn, 2015). ADVANCE is a multicenter collaborative led by the OCHIN (not an acronym) community health information network in partnership with Health Choice Network, Fenway Health, and Oregon Health & Science University. Outpatient EHR data from the three data-sharing partner organizations (HCN, Fenway, OCHIN) are integrated and standardized into a common data model (DeVoe et al., 2014). ADVANCE data partners serve federally qualified health centers and other CHCs, “safety net” clinics that provide care to medically underserved patient populations across the United States. The ADVANCE patient population spans 24 states and is demographically similar to the national profile of CHC patients (National Association of Community Health Centers, 2017).

For the current study, we included CHCs that were live on their EHR systems as of the start of the study period (January 1, 2012), provided primary and preventive care services to women, and were located in states that either expanded Medicaid under the ACA on January 1, 2014, or did not expand by the study period’s end (December 31, 2015). Medicaid expansion status was our primary independent variable; all study clinics were either in a state that expanded Medicaid on January 1, 2014, or did not expand before December 31, 2015. Wisconsin was treated as an expansion state because they opened insurance enrollment through the ACA exchange to adults up to 100% FPL (Hoopes et al., 2016; Huguet et al., 2017). Our analytic sample included 141 CHCs from 4 nonexpansion states (FL, KS, MO, and NC) and 213 CHCs from 10 expansion states (CA, HI, MA, MD, NM, OH, OR, RI, WA, and WI).

From included CHCs, we included nonpregnant females aged 11 to 65 who were eligible (or due) for at least one focal preventive service (as listed elsewhere in this article). The study population was limited to active patients, defined as a person with at least one ambulatory visit during the 2-year time period of interest. Women with any indication of pregnancy during the study were excluded because Medicaid eligibility differs for pregnant women unrelated to ACA expansion. We then looked at the proportion of women and girls who received preventive services (as a numerator) out of the number of women and girls who were eligible for them (as a denominator).

### Preventive Service Measures

We assessed prevalence of receipt of six preventive services recommended by the U.S. Preventive Services Task Force (2019) and/or Centers for Disease Control and Prevention (2019) during the study period: cervical cancer screening, chlamydia screening, human papilloma virus (HPV) vaccination, human immunodeficiency virus (HIV) screening, blood pressure screening, and influenza vaccination. At the patient level, we assessed individual eligibility in each period (before and after the ACA) based on standard definitions (see Table 2 footnotes), and among those eligible for a service, we coded a binary indicator of whether or not the service was received in the period or the appropriate look-back window.

For example, cervical cancer screening is recommended for women ages 21 to 64, either by receiving Papanicolaou (pap) testing every 3 years, or for women ages 30 to 64 receiving pap plus HPV cotesting every 5 years. For a woman who was age 32 in 2012 and had EHR documentation of pap/HPV cotesting in 2011, she is both eligible for and covered by this screening in the before and after periods, and would be included in the pre- and post-ACA implementation denominators if she had a visit in both time periods of interest. If EHR documentation indicated she had received only pap/HPV cotesting in 2011, she was considered covered in the before period but not covered for the post-period. For HPV vaccination we required one or more vaccines for patients between the ages of 11 and 26 (at any time before the end of the time period of interest); assessing series completion was not possible based on the available immunization fields in the ADVANCE database. Blood pressure screening and flu vaccination were considered complete if they occurred at least once in the 2-year period. Additional details on the eligibility and measurement of each service is provided in the footnote to Table 2.

### Covariates

We used the following patient-level covariates as collected in the EHR: age, race/ethnicity, household income as percent of FPL, and urban/rural residence. Hispanic ethnicity included all patients with Hispanic ethnicity documented as well as patients listing Spanish as their preferred spoken language (regardless of reported ethnicity). Urban/rural status was assigned using Rural-Urban Commuting Area codes (USDA, 2010) linked to patients’ zip code of last-known residence. Time-varying covariates (age, FPL, and insurance type) were assigned as of each patient’s last ambulatory visit in each of the before and after periods.

As a measure of medical complexity, we calculated the Charlson Comorbidity Index (Charlson et al., 2008) for each patient based on active problem list diagnoses at the end of each period; an enhanced version of the index was used that considers additional physical, mental, and behavioral health conditions. To control for differences in use, we computed average annual visit rates per patient period, and a binary indicator of new patient status (based on having a visit with an evaluation and management services CPT code between 99201 and 99205 or 99381 and 99387). Patients were assigned to a primary CHC facility according to the most frequently visited facility in the given period. Each patient’s primary health center was classified as expansion/nonexpansion based on the state where it was located.

CHCs are required to collect and report many individual-level demographic variables to the U.S. Health Resources and Services Administration to receive funding or designation under the Health Center Program. Therefore, the ADVANCE database contains very little missing data on patient race/ethnicity, language, and FPL. Where data elements were missing, however, we coded a separate missing/unknown category so as not to exclude these patients from our analysis.

### Analysis

We computed descriptive statistics for all patients eligible for at least one preventive service, stratified by expansion status and pre- and post-period eligibility. We calculated absolute standardized mean differences (ASMD) to assess whether the patient samples in each expansion group differed substantially between the two study periods. This balance measure is increasingly used in observational studies to compare distributional differences between groups, with extensions to binary and multinomial variables (Austin, 2009a). Importantly, ASMDs are not affected by sample size―an important feature when large sample sizes render most *p* values significant―and can be used to compare distributions between partially overlapping (i.e., non-independent) groups, as in this instance where patients may be eligible for services during one or both periods.

To make comparisons between expansion and nonexpansion groups, we used ASMDs to compare distributional differences in demographic and clinical measures. We considered an ASMD of greater than 0.1 to denote marginal differences between the groups being compared (Austin, 2009b). We calculated unadjusted and adjusted prevalence estimates of eligible women who received each preventive service in the before and after periods, by expansion status. To assess change in receipt of preventive services *within* and *between* expansion groups, we used a difference-in-differences (DID) approach (Dimick & Ryan, 2014; French & Heagerty, 2008), which compares prevalence in screening before and after ACA implementation in expansion versus nonexpansion states. Specifically, we fit generalized estimating equation (GEE) models with an identity link to obtain adjusted absolute prevalence differences comparing pre- versus post-ACA implementation changes in screening within expansion groups, and DID estimates with 95% confidence intervals (CI) to test post- versus pre-ACA implementation changes between expansion groups (Zeger, Liang, & Albert, 1988). GEE models included an indicator for ACA period (before vs. after), Medicaid expansion status, and the interaction between these variables.

GEE models were adjusted for age (except in the HPV vaccination model because it was restricted to ages 11–26), race/ethnicity, FPL, health insurance type, urban/rural status, Charlson Comorbidity Index, annual visit rate, and health system. GEE models implemented a robust sandwich standard error estimator with a working independent correlation structure to account for the clustering of patients within CHCs. This study (Cottrell et al., 2019) was approved by Western Institutional Review Board.

## Results

A total of 392,703 patients from expansion states and 318,418 patients from nonexpansion states were eligible for at least one preventive service and included in the analysis (Table 1). Women accessing care in expansion state CHCs were more commonly non-Hispanic White (48.3%), whereas the women using CHCs in nonexpansion states were more often of other racial/ethnic groups (40.2% Hispanic, 27.4% non-Hispanic Black). A large share of the women from both expansion and nonexpansion states had household incomes under the ACA Medicaid eligibility limit of 138% FPL (66.8% expansion, 75.8% nonexpansion). Women from expansion state CHCs had slightly higher levels of medical complexity (Charlson Comorbidity Index ≥3: 21.5% vs. 16.9% of nonexpansion patients), as well as higher ambulatory visit rates (mean annual visits, 2.9 expansion vs. 2.1 nonexpansion).

A greater share of women in nonexpansion states were new patients at some point in the study period (45.5% expansion vs.56.8% nonexpansion). In Medicaid expansion states, the prevalence of women without health insurance decreased by nearly one-half, from 33.9% before to 18.4% after ACA implementation, whereas the share of women covered by Medicaid increased from 37.6% to 54.3%, and those with private health insurance increased minimally from 17.2% to 19.3%. In nonexpansion states, the prevalence of women without insurance decreased less drastically, from 48.0% before to 37.6% after ACA implementation; Medicaid coverage rates changed very little; and the share of women privately insured increased more significantly from 7.3% to 19.7%.

Within the Medicaid expansion groups, the populations in the pre- and post-ACA implementation samples did not differ demonstrably on any measures (all ASMD <0.1) except insurance status (ASMD ~0.4 for both expansion and nonexpansion groups). Between expansion and nonexpansion groups, the total study populations differed significantly on all demographic and clinical measures (*p* < .001 for all; ASMD >0.1 for all except continuous age; Table 1).

The unadjusted and adjusted screening prevalences for expansion groups and period are reported in Table 2. Across the entire sample, the prevalence of receipt of each preventive service, as well as before and after ACA implementation adjusted absolute prevalence differences, increased across all preventive services (except blood pressure screening, which remained high throughout the study) between pre- and post-ACA implementation time periods―a pattern observed in both expansion and nonexpansion states.

Cervical cancer screening in expansion state CHCs increased 6.7 percentage points, from 45.1% before to 51.7% after ACA implementation. The increase in screening within nonexpansion state CHCs was smaller (4.8 percentage points, from 47.0% to 51.7%). When adjusted for covariates, the pre–post ACA implementation difference was not statistically different between Medicaid expansion and nonexpansion groups (adjusted DID estimate, 1.45 percentage points; 95% CI, −0.50 to 3.41). The adjusted absolute prevalence difference of 1.45 means that cervical cancer screening was estimated to increase 1.45 percentage points more before to after ACA implementation among Medicaid expansion states compared with nonexpansion states. Sizable gains were also seen in HPV vaccination and HIV screening rates across all states, but without significant differences by expansion status.

Chlamydia screening increased significantly more before to after ACA implementation in nonexpansion states relative to expansion states (expansion increase, 5.9%; nonexpansion increase, 10.4%; adjusted DID, −4.21; 95% CI, −6.98 to −1.45). The absolute prevalence difference of −4.21 means that chlamydia screening was estimated to increase 4.21 percentage points more before to after ACA implementation among Medicaid nonexpansion states compared with expansion states.

After adjustment, influenza vaccination was estimated to increase by 1.98 percentage points more among expansion states compared with nonexpansion states (95% CI, 0.91–3.05), which was statistically significant. Blood pressure screening remained high before and after ACA implementation and differed very little between Medicaid expansion and nonexpansion groups. The adjusted absolute prevalence difference was statistically significant (adjusted DID, 1.55; 95% CI, 0.51–2.60).

## Discussion

Among women and girls receiving care in CHCs, receipt of five of six key preventive services (cervical cancer screening, HPV vaccination, HIV screening, chlamydia screening, and influenza vaccination) increased after implementation of the ACA in both Medicaid expansion and nonexpansion states. (Blood pressure screening did not increase after ACA implementation among Medicaid nonexpansion states but was already very high before the ACA and remained high in both expansion and nonexpansion groups.) These findings are consistent with previous literature that suggests other preventive services like mammography and colorectal cancer screening increased overall after the implementation of the ACA (Alharbi et al., 2019; Huguet et al., 2019; Sun et al., 2018). Gains in preventive service delivery have previously been attributed to broad insurance expansion (both public and private) (Huguet et al., 2017), development of Accountable Care Organizations with incentivized focus on quality metrics (although the proliferation of Accountable Care Organizations was underway before Medicaid expansion in 2014) (Meyer et al., 2017), and additional resources for safety net health centers through federal grants and increased health insurance revenue (Han et al., 2017; Huguet et al., 2019).

Contrary to our hypothesis, we observed minimal differences between Medicaid expansion and nonexpansion states in the magnitude of improvement in receipt of recommended preventive care after ACA implementation. With the exception of influenza vaccination and blood pressure screening, which increased in expansion states more than nonexpansion states, and chlamydia screening, which increased more in nonexpansion state CHCs than in expansion states, change in receipt of preventive services did not differ significantly between expansion and nonexpansion states.

Previous studies comparing service delivery in Medicaid expansion versus nonexpansion states have been mixed (Antonisse et al., 2019; Huguet et al., 2018), which suggests that multilevel factors outside of Medicaid expansion alone―such as increased coverage of preventive services―may impact overall receipt of preventive services. In this study, the total amount of change may have also been impacted by factors such as initial screening prevalence (e.g., the initial rate of chlamydia screening was lower among nonexpansion states) and differential EHR capture of data (e.g., state-level data quality regarding when and where vaccinations are received and whether the CHC’s EHR can directly access that information may particularly impact the measured rate of influenza vaccination, which is commonly given outside the CHC) (Centers for Disease Control and Prevention, 2018). Although differences in blood pressure screening rates were statistically different between Medicaid expansion and nonexpansion states before and after ACA implementation, the very small magnitude of this difference with the very high screening rates throughout the study (>95%) suggest little clinical significance of this difference.

Overall, receipt of preventive services among female CHC patients remained low despite broad improvements that were observed after implementation of the ACA and were present among both Medicaid expansion and nonexpansion states. Still, with the exception of the rate of flu vaccination in nonexpansion states (see the Limitations section), the observed prevalence of receipt of individual preventive services was roughly similar to other studies measuring preventive care in this population (Cowburn et al., 2013; Huguet et al., 2019; Walker et al., 2018), although they lag far behind aspirational metrics such as Healthy People 2020 (Office of Disease Prevention and Health Promotion, 2014). Reasons for a lack of consistent differences in pre-/post-ACA implementation delivery of services between Medicaid expansion and nonexpansion states are likely multifactorial and require further exploration in additional states and with additional types of services.

### Limitations

Although the use of objective clinical data was a strength of this study and enabled highly accurate data free from recall bias, EHR data were limited to our network. Any preventive care received outside CHCs in the ADVANCE network would not have been captured, resulting in potentially under-reported receipt of services. Undercapture of services is particularly likely for influenza vaccination, which is widely available outside of traditional clinical settings, and HPV vaccination, which may have been received in the remote past and thus at greater risk of not being documented. If care were received elsewhere, it would result in a falsely low estimate of preventive care received, though previous study of this particular CHC population demonstrates a very low rate of attrition, even when patients undergo insurance change (Huguet et al., 2020). As a study limited to active patients with a visit during the study period, the study was unable to observe individuals who did not use care during that period, so the results reflect receipt of preventive services only among individuals who utilize health care at CHCs. We had limited data availability on some of our outcome measures from certain reporting clinics (e.g., one health system does not submit immunization data to ADVANCE and another does not use standard LOINC or CPT codes for laboratory data); thus, a small subset of the clinics were excluded from the immunization and chlamydia measures. Some modifications to standard measure specifications were necessary to better align with EHR data (e.g., for chlamydia screening, a patient’s sexual activity was presumed because this information is not consistently documented in the EHR), and all outcomes were measured across 2-year periods even if they are recommended annually.

Although data from this study came from one of the largest national networks of CHCs and included CHC data from 14 states, these CHCs are not representative of the population of patients within their states. There is also a potential for unmeasured confounding from rapidly changing state-level policies (other than Medicaid expansion) as well as regional norms of practice that were beyond the scope of this study. Finally, as a study of individual patients who sought care at CHCs (not the population at large), the impact of changing patient-panel composition on the prevalence of receipt of preventive care is uncertain. That is, when the population has better access to care, the population of patients (and therefore the denominator in this type of study) changes and may lead to bias despite rigorous demographic adjustments used in this study. This limitation has been noted elsewhere (Allen & Sommers, 2019), and although it provides important context for understanding the population-level impact of ACA implementation, the observed changes in patient- and clinic-level prevalence of receipt of preventive care remain important to clinical staff tasked with providing this care, as well as policymakers interested in developing systems to overcome remaining barriers to care.

### Implications for Practice and/or Policy

Despite observed improvements in preventive service delivery between pre- and post-ACA implementation time points, the persistent low prevalence of receipt of key preventive services highlights the need for continued efforts to address barriers to preventive care among women and girls who seek care in CHCs. Beyond access to health care, patients served by CHCs may experience many additional barriers to receipt of preventive care, including factors measured (e.g., race/ethnicity, income, number of visits) and unmeasured (e.g., health literacy, transportation, neighborhood characteristics, history of trauma) by this study. Further understanding the barriers and facilitators of preventive care is needed, as are urgent intervention efforts to improve receipt of critical preventive services. In addition to reflecting the needs of the safety net specifically, this study adds to a larger body of research demonstrating suboptimal use of preventive services in the US. This low use has persisted in some populations even when financial barriers are removed (Cross-Barnet, Colligan, McNeely, Strawbridge, & Lloyd, 2019; Misra, Lloyd, Strawbridge, & Wensky, 2018), suggesting the need for systemic changes to align priorities, support patients, and enable clinicians and health care teams to more effectively deliver preventive care (Yarnall, Pollak, Østbye, Krause, & Michener, 2003).

## Conclusions

Receipt of key preventive services among female CHC patients increased after ACA implementation in both Medicaid expansion and nonexpansion states. There were no consistent trends toward greater improvement within Medicaid expansion (versus nonexpansion) states. Despite overall improvements, the receipt of preventive services remains low, highlighting the need for additional support of the CHC system to improve preventive service delivery to women and girls.

## Figures and Tables

**Table 1:** Demographic and Health Care Use Characteristics of Eligible Female Patients, before and after the ACA, by Medicaid Expansion Status

Patient Characteristics	Expansion States			Nonexpansion States			ASMD (Total)
	Total, *N* (%)	Pre-ACA, *n* (%)	Post-ACA, *n* (%)	Total, *N* (%)	Pre-ACA, *n* (%)	Post-ACA, *n* (%)	
*N* eligible	392,703	268,967	270,868	318,418	200,354	208,190	
Age (Y)							
Mean ± SD	37.7 ± 14.9	37.2 ± 14.8	37.6 ± 14.8	38.5 ± 15.3	37.9 ± 15.1	38.4 ± 15.3	0.055
12–26	110,440 (28.1)	78,434 (29.2)	76,757 (28.3)	87,213 (27.4)	57,122 (28.5)	57,019 (27.4)	0.109
27–39	112,070 (28.5)	73,102 (27.2)	74,162 (27.4)	79,825 (25.1)	48,205 (24.1)	50,374 (24.2)	
≥40	170,193 (43.3)	117,431 (43.7)	119,949 (44.3)	151,380 (47.5)	95,027 (47.4)	100,797 (48.4)	
Race/ethnicity							0.585
Hispanic	131,926 (33.6)	93,097 (34.6)	94,824 (35.0)	128,073 (40.2)	78,151 (39.0)	85,495 (41.1)	
Non-Hispanic White	189,657 (48.3)	128,622 (47.8)	127,824 (47.2)	90,058 (28.3)	56,616 (28.3)	57,094 (27.4)	
Non-Hispanic Black	39,909 (10.2)	28,079 (10.4)	27,125 (10.0)	87,267 (27.4)	58,119 (29.0)	57,182 (27.5)	
Non-Hispanic other	23,112 (5.9)	14,665 (5.5)	15,938 (5.9)	6,970 (2.2)	4,002 (2.0)	4,835 (2.3)	
Unknown	8,099 (2.1)	4,504 (1.7)	5,157 (1.9)	6,050 (1.9)	3,466 (1.7)	3,584 (1.7)	
Federal poverty level							0.261
≤138%	262,494 (66.8)	184,899 (68.7)	183,380 (67.7)	241,457 (75.8)	156,446 (78.1)	157,306 (75.6)	
>138%	54,295 (13.8)	37,326 (13.9)	38,964 (14.4)	44,841 (14.1)	26,423 (13.2)	30,658 (14.7)	
Unknown	75,914 (19.3)	46,742 (17.4)	48,524 (17.9)	32,120 (10.1)	17,485 (8.7)	20,226 (9.7)	
Urban/rural							0.273
Rural	27,189 (6.9)	19,511 (7.3)	18,495 (6.8)	10,478 (3.3)	7,881 (3.9)	7,812 (3.8)	
Urban	347,838 (88.6)	238,557 (88.7)	239,189 (88.3)	307,071 (96.4)	191,866 (95.8)	199,930 (96.0)	
Missing	17,676 (4.5)	10,899 (4.1)	13,184 (4.9)	869 (0.3)	607 (0.3)	448 (0.2)	
Charlson score							
Mean ± SD	1.3 ± 2.0	1.3 ± 2.0	1.4 ± 2.0	1.1 ± 1.8	1.1 ± 1.8	1.1 ± 1.9	0.107
0	217,705 (55.4)	149,939 (55.7)	142,906 (52.8)	187,926 (59.0)	119,032 (59.4)	116,791 (56.1)	0.137
1–2	90,682 (23.1)	62,481 (23.2)	67,001 (24.7)	76,566 (24.0)	47,726 (23.8)	54,911 (26.4)	
3–4	52,249 (13.3)	35,668 (13.3)	37,255 (13.8)	35,093 (11.0)	22,109 (11.0)	23,251 (11.2)	
≥5	32,067 (8.2)	20,879 (7.8)	23,706 (8.8)	18,833 (5.9)	11,487 (5.7)	13,237 (6.4)	
Annual ambulatory visit rate							
Mean (SD)	2.9 (3.7)	2.7 (3.4)	2.8 (3.5)	2.1 (2.5)	2.1 (2.3)	2.0 (2.1)	0.267
≤1	147,038 (37.4)	107,361 (39.9)	104,621 (38.6)	152,390 (47.9)	97,652 (48.7)	103,196 (49.6)	0.305
>1–4	167,906 (42.8)	111,093 (41.3)	114,461 (42.3)	130,172 (40.9)	79,283 (39.6)	82,919 (39.8)	
>4–7	45,696 (11.6)	32,195 (12.0)	33,452 (12.3)	24,767 (7.8)	17,461 (8.7)	16,945 (8.1)	
>7	32,063 (8.2)	18,318 (6.8)	18,334 (6.8)	11,089 (3.5)	5,958 (3.0)	5,130 (2.5)	
New patient status							0.557
Yes	178,799 (45.5)	90,671 (33.7)	91,190 (33.7)	180,718 (56.8)	89,670 (44.8)	95,566 (45.9)	
No	213,904 (54.5)	178,296 (66.3)	179,678 (66.3)	137,700 (43.2)	110,684 (55.2)	112,624 (54.1)	
Insurance							0.557
Medicaid	186,812 (47.6)	100,999 (37.6)	146,982 (54.3)	83,812 (26.3)	52,497 (26.2)	57,368 (27.6)	
Medicare/other public	34,646 (8.8)	30,378 (11.3)	21,792 (8.0)	52,927 (16.6)	37,109 (18.5)	31,419 (15.1)	
Private	72,911 (18.6)	46,396 (17.2)	52,241 (19.3)	48,041 (15.1)	14,574 (7.3)	41,061 (19.7)	
Uninsured	98,334 (25.0)	91,194 (33.9)	49,853 (18.4)	133,638 (42.0)	96,174 (48.0)	78,342 (37.6)	

*Abbreviation*: ASMD, absolute standardized mean difference.

New patient status defined as ever/never new patient in period. All other time-varying characteristics assessed as of last visit in period.

The ASMD compares the total expansion vs. total nonexpansion distributions. All within-expansion group ASMDs comparing pre- versus post-ACA eligible populations were <0.10 except insurance type (expansion ASMD = 0.398, nonexpansion ASMD = 0.414; data not shown).

All between-group *p* values were significant, *p* < .001 (data not shown).

**Table 2:** Preventive Service Receipt Among Eligible Female Patients, before versus after the ACA, by Medicaid Expansion Status

Preventive Services	Expansion States			Nonexpansion States			Absolute Prevalence DID, before vs. after the ACA, Expansion vs. Nonexpansion (95% CI)	*p* Value, DID Estimator
	Pre-ACA (2012–2013)	Post-ACA (2014–2015)	Absolute Prevalence Difference, before vs. after the ACA (95% CI)	Pre-ACA (2012–2013)	Post-ACA (2014–2015)	Absolute Prevalence Difference, before vs. after the ACA (95% CI)		
Cervical cancer screening*								
*N* eligible	203,144	207,916		154,200	161,316			
Screening prevalence, crude prevalence difference	45.1%	51.7%	6.56 (5.20 to 7.91)	47.0%	51.7%	4.75 (2.97 to 6.54)	1.81 (−0.44 to 4.05)	.114
Adjusted absolute prevalence difference			6.50 (5.28 to 7.71)			5.04 (3.52 to 6.56)	1.45 (−0.50 to 3.41)	.144
Chlamydia screening†								
*N* eligible	31,814	30,809		22,519	21,594			
Screening prevalence, crude prevalence difference	40.8%	44.4%	3.52 (2.02 to 5.02)	35.2%	42.1%	6.92 (4.50 to 9.33)	−3.40 (−6.24 to −0.55)	.019
Adjusted absolute prevalence difference			6.71 (4.93 to 8.48)			10.92 (8.32 to 13.52)	−4.21 (−6.98 to −1.45)	**.003**
HPV vaccination‡								
*N* eligible	75,791	73,831		57,562	57,358			
Vaccination prevalence, crude prevalence difference	39.1%	47.7%	8.63 (7.50 to 9.76)	22.1%	29.0%	6.85 (5.27 to 8.43)	1.79 (−0.16 to 3.73)	.072
Adjusted absolute prevalence difference			8.30 (7.28 to 9.32)			6.60 (5.15 to 8.06)	1.70 (−0.09 to 3.38)	.062
HIV screening§								
*N* eligible	231,751	236,379		167,946	175,274			
Screening prevalence, crude prevalence difference	17.4%	23.6%	6.12 (5.20 to 7.04)	20.6%	27.2%	6.51 (4.41 to 8.61)	−0.39 (−2.68 to 1.90)	.740
Adjusted absolute prevalence difference			4.97 (4.16 to 5.79)			6.01 (3.91 to 8.11)	−1.04 (−3.30 to 1.22)	.367
Blood pressure screening∥								
*N* eligible	232,092	237,266		169,750	177,386			
Screening prevalence, crude prevalence difference	95.5%	96.0%	0.54 (−0.23 to 1.31)	96.7%	95.7%	−0.95 (−1.67 to −0.22)	1.48 (0.43 to 2.54)	.006
Adjusted absolute prevalence difference			0.76 (−0.03 to 1.54)			−0.80 (−1.51 to −0.08)	1.55 (0.51 to 2.60)	**.004**
Flu vaccination¶								
*N* eligible	262,260	264,410		201,185	209,440			
Vaccination prevalence, crude prevalence difference	30.8%	34.5%	3.70 (2.81 to 4.59)	12.6%	14.1%	1.51 (0.77 to 2.25)	2.19 (1.03 to 3.34)	<.001
Adjusted absolute prevalence difference			3.67 (2.85 to 4.49)			1.69 (1.00 to 2.37)	1.98 (0.91 to 3.05)	**<.001**

*Abbreviation*: ACA, Affordable Care Act; DID, difference-in-difference; HPV, human papilloma virus.

Values in bold significant at *p* ≤ .005.

Difference estimates obtained from generalized estimating equation models clustered by primary clinic, assuming an independent correlation structure.

* Pap within 3 years among women 21–64, or pap + HPV testing within 5 years among women 30–64. Women with indication of hysterectomy or personal history of cervical cancer excluded. Model adjusted for age, federal poverty level, race/ethnicity, urban/rural status, Charlson comorbidity index, annual visit rate, new patient status in period, and health system.

† Chlamydia/gonorrhea screening among women 19–24. Sexually active criteria assumed. Limited to facilities reporting standard codes (CPT and LOINC) sufficient for measure ascertainment. Model adjusted for age in years, federal poverty level, race/ethnicity, urban/rural status, Charlson comorbidity index, annual visit rate, new patient status in period, and health system.

‡ HPV vaccination ever (≥1 in series) among girls/women 11–26. Limited to facilities reporting immunization data. Model adjusted for federal poverty level, race/ethnicity, urban/rural status, Charlson comorbidity index, annual visit rate, new patient status in period, and health system.

§ HIV screening ever among women 18–65. Women previously diagnosed with HIV excluded. Model adjusted for age in years, federal poverty level, race/ethnicity, urban/rural status, Charlson comorbidity index, annual visit rate, new patient status in period, and health system.

∥ Blood pressure screening among women 18–65. Model adjusted for age in years, federal poverty level, race/ethnicity, urban/rural status, Charlson comorbidity index, annual visit rate, new patient status in period, and health system.

¶ Flu vaccination among all girls/women 11–65. Limited to facilities reporting immunization data. Model adjusted for age in years, federal poverty level, race/ethnicity, urban/rural status, Charlson comorbidity index, annual visit rate, new patient status in period, and health system.

## References

[R1] Agency for Healthcare Research and Quality. (2015). ACA-covered preventive health services for women. Available: www.ahrq.gov/ncepcr/tools/healthier-pregnancy/fact-sheets/preventive-health-services.html. Accessed: November 21, 2019.

[R2] Alharbi AG, Khan MM, Horner R, Brandt H, & Chapman C (2019). Impact of Medicaid coverage expansion under the Affordable Care Act on mammography and pap tests utilization among low-income women. PLoS One, 14(4), e0214886. 30943249 10.1371/journal.pone.0214886 PMC6447234

[R3] Allen H, & Sommers BD (2019). Medicaid expansion and health: Assessing the evidence after 5 years. JAMA. [Epub ahead of print]. 10.1001/jama.2019.12345 31490532

[R4] Angier H, Hoopes M, Gold R, Bailey SR, Cottrell EK, Heintzman J,…DeVoe JE (2015). An early look at rates of uninsured safety net clinic visits after the Affordable Care Act. Annals of Family Medicine, 13(1), 10–16. 25583886 10.1370/afm.1741 PMC4291259

[R5] Angier H, Hoopes M, Marino M, Huguet N, Jacobs EA, Heintzman J,…DeVoe JE (2017). Uninsured primary care visit disparities under the Affordable Care Act. Annals of Family Medicine, 15(5), 434–442. 28893813 10.1370/afm.2125 PMC5593726

[R6] Antonisse L, Garfield R, Rudowitz R, & Guth M (2019). The effect of Medicaid expansion under the ACA: Updated findings from a literature review. Available: http://files.kff.org/attachment/Issue-brief-The-Effects-of-Medicaid-Expansion-under-the-ACA-Findings-from-a-Literature-Review. Accessed: November 13, 2019.

[R7] Austin P (2009a). Balance diagnostics for comparing the distribution of baseline covariates between treatment groups in propensity-score matched samples. Statistics in Medicine, 28, 3083–3107. 19757444 10.1002/sim.3697 PMC3472075

[R8] Austin P (2009b). Using the standardized difference to compare the prevalence of a binary variable between two groups in observational research. Communications in Statistics - Simulation and Computation, 38, 1228–1234.

[R9] Bailey SR, O’Malley JP, Gold R, Heintzman J, Marino M, & DeVoe JE(2015). Receipt of diabetes preventive services differs by insurance status at visit. American Journal of Preventive Medicine, 48(2), 229–233. 25442228 10.1016/j.amepre.2014.08.035 PMC4301980

[R10] Centers for Disease Control and Prevention. (2018). Estimates of influenza vaccine coverage among adults - United States, 2017–18 Flu Season. Available: www.cdc.gov/flu/fluvaxview/coverage-1718estimates.htm. Accessed: November 13, 2019.

[R11] Centers for Disease Control and Prevention. (2019). Immunization schedules for health care providers. Available: www.cdc.gov/vaccines/schedules/index.html. Accessed: November 13, 2019.

[R12] Charlson ME, Charlson RE, Peterson JC, Marinopoulos SS, Briggs WM, & Hollenberg JP (2008). The Charlson comorbidity index is adapted to predict costs of chronic disease in primary care patients. Journal of Clinical Epidemiology, 61(12), 1234–1240. 18619805 10.1016/j.jclinepi.2008.01.006

[R13] Corley D, Feigelson H, Lieu T, & McGlynn E (2015). Building data infrastructure to evaluate and improve quality: PCORnet. Journal of Oncology Practice, 11, 204–206. 25980016 10.1200/JOP.2014.003194 PMC4438109

[R14] Cottrell E, Darney BG, Marino M, Templeton AR, Jacob L, Hoopes M,…Hatch B (2019). Study protocol: A mixed-methods study of women’s healthcare in the safety net after Affordable Care Act implementation - EVERYWOMAN. Health Research Policy and Systems, 17(1), 58. 31186028 10.1186/s12961-019-0445-y PMC6558747

[R15] Courtemanche C, Marton J, Ukert B, Yelowitz A, & Zapata D (2018). Effects of the Affordable Care Act on health care access and self-assessed health after 3 years. Inquiry, 55. 46958018796361–46958018796361. 30188235 10.1177/0046958018796361 PMC6146333

[R16] Cowburn S, Carlson MJ, Lapidus JA, & DeVoe JE (2013). The association between insurance status and cervical cancer screening in community health centers: Exploring the potential of electronic health records for population-level surveillance, 2008–2010. Preventing Chronic Disease, 10, E173. 24157076 10.5888/pcd10.130034 PMC3809921

[R17] Cross-Barnet C, Colligan EM, McNeely J, Strawbridge LM, & Lloyd JT (2019). Facilitators and barriers to optimal preventive service use among providers and older patients. Geriatric Nursing, 40(1), 72–77. 30122404 10.1016/j.gerinurse.2018.06.017

[R18] Department of Health and Human Services (2017). Fiscal year 2017 health resources and services administration, justification of estimates for appropriations committees. Available: www.hrsa.gov/sites/default/files/about/budget/budgetjustification2017.pdf. Accessed: November 13, 2019.

[R19] DeVoe JE, Fryer GE, Phillips R, & Green L (2003). Receipt of preventive care among adults: Insurance status and usual source of care. American Journal of Public Health, 93(5), 786–791. 12721145 10.2105/ajph.93.5.786 PMC1447840

[R20] DeVoe JE, Gold R, Cottrell E, Bauer V, Brickman A, Puro J, … Fields S (2014). The ADVANCE network: Accelerating data value across a national community health center network. Journal of the American Medical Informatics Association, 21(4), 591–595. 24821740 10.1136/amiajnl-2014-002744 PMC4078289

[R21] Dimick JB, & Ryan AM (2014). Methods for evaluating changes in health care policy: The difference-in-differences approach. JAMA, 312(22), 2401–2402. 25490331 10.1001/jama.2014.16153

[R22] French B, & Heagerty PJ (2008). Analysis of longitudinal data to evaluate a policy change. Statistics in Medicine, 27(24), 5005–5025. 18618416 10.1002/sim.3340 PMC3415557

[R23] Hall KS, Fendrick AM, Zochowski M, & Dalton VK (2014). Women’s health and the Affordable Care Act: High hopes versus harsh realities? American Journal of Public Health, 104(8), e10–e13. 10.2105/AJPH.2014.302045 PMC4103253 24922171

[R24] Han X, Luo Q, & Ku L (2017). Medicaid Expansion And Grant Funding Increases Helped Improve Community Health Center Capacity. Health Affairs (Millwood), 36(1), 49–56. 10.1377/hlthaff.2016.0929 28069846

[R25] Hatch B, Smith N, McBurnie MA, Quach T, Mayer KH, Dunne MJ, & Cottrell E (2018). Impacts of the Affordable Care Act on Community Health Centers: Characteristics of new patients and early changes in delivery of care. Journal of Ambulatory Care Management, 41(4), 250–261. 29771741 10.1097/JAC.0000000000000244

[R26] Hoopes MJ, Angier H, Gold R, Bailey SR, Huguet N, Marino M, & DeVoe JE (2016). Utilization of community health centers in Medicaid expansion and nonexpansion states, 2013–2014. Journal of Ambulatory Care Management, 39(4), 290–298. 26765808 10.1097/JAC.0000000000000123 PMC4942402

[R27] Huguet N, Angier H, Rdesinski R, Hoopes M, Marino M, Holderness H, & DeVoe JE (2019). Cervical and colorectal cancer screening prevalence before and after Affordable Care Act Medicaid expansion. Preventive Medicine, 124, 91–97. 31077723 10.1016/j.ypmed.2019.05.003 PMC6578572

[R28] Huguet N, Hoopes MJ, Angier H, Marino M, Holderness H, & DeVoe JE (2017). Medicaid expansion produces long-term impact on insurance coverage rates in community health centers. Journal of Primary and Care Community Health, 8(4), 206–212. 10.1177/2150131917709403 PMC5665709 28513249

[R29] Huguet N, Kaufmann J, O’Malley J, Angier H, Hoopes M, DeVoe JE, & Marino M (2020). Using electronic health records in longitudinal studies: Estimating patient attrition. Medical Care, 58(Suppl 6), S46–S52. 32412953 10.1097/MLR.0000000000001298 PMC7365658

[R30] Huguet N, Springer R, Marino M, Angier H, Hoopes M, Holderness H, & DeVoe JE (2018). The Impact of the Affordable Care Act (ACA) Medicaid expansion on visit rates for diabetes in safety net health centers. Journal of the American Board of Family Medicine, 31(6), 905–916. 30413546 10.3122/jabfm.2018.06.180075 PMC6329010

[R31] Kaiser Family Foundation. (2018). Proposed changes to Title X: Implications for women and family planning providers. Available: www.kff.org/womens-health-policy/issue-brief/proposed-changes-to-title-x-implications-for-women-and-family-planning-providers/. Accessed: November 21, 2019.

[R32] Kaiser Family Foundation. (2020). Status of state action on the Medicaid expansion decision. Available: http://kff.org/health-reform/state-indicator/state-activity-around-expanding-medicaid-under-the-affordable-care-act/. Accessed: November 13, 2019.

[R33] Kominski GF, Nonzee NJ, & Sorensen A (2017). The Affordable Care Act’s impacts on access to insurance and health care for low-income populations. Annual Review of Public Health, 38, 489–505. 10.1146/annurev-publhealth-031816-044555 PMC5886019 27992730

[R34] Maciosek MV, Coffield AB, Flottemesch TJ, Edwards NM, & Solberg LI (2010). Greater use of preventive services in U.S. health care could save lives at little or no cost. Health Affairs (Millwood), 29(9), 1656–1660. 10.1377/hlthaff.2008.0701 20820022

[R35] Marino M, Bailey SR, Gold R, Hoopes MJ, O’Malley JP, Huguet N, … DeVoe JE (2016). Receipt of preventive services after Oregon’s randomized Medicaid experiment. American Journal of Preventive Medicine, 50(2), 161–170. 26497264 10.1016/j.amepre.2015.07.032 PMC4718854

[R36] Meyer CP, Krasnova A, Sammon JD, Lipsitz SR, Weissman JS, Sun M, & Trinh QD (2017). Accountable care organizations and the use of cancer screening. Preventive Medicine, 101, 15–17. 28528171 10.1016/j.ypmed.2017.05.017

[R37] Misra A, Lloyd JT, Strawbridge LM, & Wensky SG (2018). Use of welcome to Medicare visits among older adults following the Affordable Care Act. American Journal of Preventive Medicine, 54(1), 37–43. 29132952 10.1016/j.amepre.2017.08.030

[R38] National Association of Community Health Centers. (2017). Community Health Center Chartbook, June 2017. Available: www.nachc.org/wp-content/uploads/2017/06/Chartbook2017.pdf. Accessed: November 13, 2019.

[R39] Office of Disease Prevention and Health Promotion (2014). HealthyPeople.gov.Available: www.healthypeople.gov/. Accessed: November 13, 2019.

[R40] Pazol K, Robbins CL, Black LI, Ahrens KA, Daniels K, Chandra A, … Gavin LE (2017). Receipt of selected preventive health services for women and men of reproductive age - United States, 2011–2013. MMWR Surveillence Summaries, 66(20), 1–31. 10.15585/mmwr.ss6620a1 PMC5879726 29073129

[R41] Sabik LM, & Adunlin G (2017). The ACA and cancer screening and diagnosis. Cancer Journal (Sudbury, Mass.), 23(3), 151–162. 10.1097/PPO.0000000000000261 PMC5445928 28537960

[R42] Stolp H, & Fox J (2015). Increasing receipt of women’s preventive services. Journal of Women’s Health (2002), 24(11), 875–881. 10.1089/jwh.2015.5552 PMC4643365 26447836

[R43] Sun M, Cole AP, Lipsitz SL, & Trinh Q-D (2018). Trends in breast, colorectal, and cervical cancer incidence following the Affordable Care Act: Implications for cancer screeningtrends in cancer incidence following the Affordable Care Act letters. JAMA Oncology, 4(1), 128–129. 29098266 10.1001/jamaoncol.2017.3861 PMC5833652

[R44] Tilhou AS, Huguet N, DeVoe J, & Angier H (2020). The Affordable Care Act Medicaid expansion positively impacted community health centers and their patients. Journal of General Internal Medicine, 34, 1292–1295. 10.1007/s11606-019-05571-w PMC7174462 31898120

[R45] U.S. Government. (2015). Coverage of certain preventive services under the Affordable Care Act. (41318). Federal Register: Government Publishing Office. Available: www.govinfo.gov/content/pkg/FR-2015-07-14/pdf/2015-17076.pdf. Accessed: November 21, 2019.

[R46] U.S. Preventive Services Task Force. (2019). USPSTF A and B recommendations. Available: www.uspreventiveservicestaskforce.org/Page/Name/uspstf-a-and-b-recommendations/. Accessed: November 21, 2019.

[R47] USDA. (2010). Rural urban commuting area codes. Available: www.ers.usda.gov/data-products/rural-urban-commuting-area-codes.aspx. Accessed: November 13, 2019.

[R48] Walker TY, Elam-Evans LD, Yankey D, Markowitz LE, Williams CL, Mbaeyi S, & Fredo B (2018). National, regional, state, and selected local area vaccination coverage among adolescents aged 13–17 years ― United States, 2017. Morbidity and Mortality Weekly Report, 67, 909–917. 30138305 10.15585/mmwr.mm6733a1 PMC6107323

[R49] Wood S, Goldberg DG, Beeson T, Bruen BK, Johnson K, Mead H, … Rosenbaum SJ (2013). Health centers and family planning: Results of a nationwide study. Available: https://hsrc.himmelfarb.gwu.edu/sphhs_policy_facpubs/60/. Accessed: November 21, 2019.

[R50] Yarnall KS, Pollak KI, Østbye T, Krause KM, & Michener JL (2003). Primary care: Is there enough time for prevention? American Journal of Public Health, 93(4), 635–641. 12660210 10.2105/ajph.93.4.635 PMC1447803

[R51] Zeger SL, Liang KY, & Albert PS (1988). Models for longitudinal data: A generalized estimating equation approach. Biometrics, 44(4), 1049–1060. 3233245

